# Quants and poets: two dimensions of MBA performance, aptitudes, and interests

**DOI:** 10.3389/fpsyg.2025.1671351

**Published:** 2026-01-13

**Authors:** Aaron S. Wallen, Zachariah C. Brown, Michael W. Morris

**Affiliations:** 1 School of Professional Studies, Columbia University, New York, NY, United States; 2 Department of Management, Hong Kong University of Science and Technology, Hong Kong, Hong Kong SAR, China; 3 Columbia Business School, New York, NY, United States

**Keywords:** MBA performance, aptitude and interests, leadership assessment, admissions predictors, social intelligence

## Abstract

**Introduction:**

Research on MBA student performance typically relies on GPA as the primary indicator of success. However, business schools aim to develop future leaders for diverse career paths, which value multiple forms of performance. We examine whether performance is better understood as multidimensional, testing a longstanding distinction in MBA discourse between “poets” and “quants.” We also examine how different forms of admissions data (i.e. standardized test scores, undergraduate grades, stated interests, and pre-MBA experiences) predict distinct forms of success.

**Methods:**

We report results from two large-N studies using survey and archival data from an elite U.S. MBA program. Study 1 examines whether core course grades reflect multiple dimensions of academic performance and whether admissions-time aptitude measures differentially predict those dimensions. Study 2 replicates these findings using archival academic, extracurricular, and peer-evaluation records and extends the analysis to leadership outcomes. Confirmatory factor analysis and multivariate regression models are used across both studies.

**Results:**

Across both studies, MBA academic performance bifurcates into two weakly correlated dimensions: systematizing (quantitative, analytical success) and social (verbal, interpersonal success). These align with the popular MBA “poet vs. quant” distinction. Quantitative aptitude predicts quantitative academic performance, whereas verbal and writing aptitude predict social academic performance. Beyond grades, social performance is uniquely associated with leadership success, including both objective attainment (e.g., club leadership roles) and peer perceptions (e.g., assertiveness and inclusiveness). Student interests further differentiate outcomes: quant-oriented interests predict quantitative academic success but negatively predict leadership attainment, whereas poet-oriented interests positively predict leadership outcomes.

**Discussion:**

These findings demonstrate that MBA success is fundamentally multidimensional and that different admissions indicators predict different forms of performance, with implications for talent assessment, leadership development, and MBA admissions practice.

## 1 Introduction

Business schools search for students who will succeed in MBA programs. But what constitutes *success* and how can schools predict it? Research on MBA student performance typically operationalizes student “success” as overall grades—typically, first-year GPA. However, schools themselves have a more variegated picture of success. Administrators want MBA students who contribute in many kinds of classes and in extra-curricular programs, such as student boards and clubs. Similarly, MBA students assess their peers along multiple dimensions, not just overall GPA. A longstanding folk construct among MBAs is the distinction between “quants” and “poets.” Quants succeed with numbers and formal systems. Poets succeed with words and people. Overall, a unitary measure like GPA may fail to capture what success really means in MBA programs.

The current research program addresses two objectives. First, we investigate the reality of the quants-poets distinction in MBA talent as it relates to performance. Second, we isolate separate admissions metrics useful in *predicting* distinct measures of performance. As in past research, we start with academic grades as measures of MBA performance and aptitude tests as measures of ability. We demonstrate that grades across the core classes fall onto two dimensions of performance, namely poet and quant. We find support that these two dimensions are also differentially predicted by corresponding poet and quant dimensions of ability. We then extend our inquiry beyond grades by incorporating measures of extra-curricular success in student organizations. Likewise, we incorporate additional success predictors beyond aptitudes, such as pre-existing interests and experiences. With this broader set of predictor and outcome measures, we find consistent support for a two-dimensional conception of MBA talent.

### 1.1 Dimensions of aptitude

The most influential theories of aptitude or mental ability come from early research on intelligence tests. Early work debated whether intelligence falls on one primary dimension or several dimensions. Spearman (1904) argued that a single dimension of test performance, general intelligence, predicted performance on a wide range of tasks. General intelligence has indeed been found to predict employee, managerial, and academic performance at various levels (Judge et al., 2004).

Thorndike (1920) later proposed a dimension of social intelligence distinct from abstract cognitive intelligence. Researchers working with verbal tests struggled to demonstrate social intelligence’s distinctiveness (Guilford, 1967; Hunt, 1928; Hendricks et al., 1969; O’Sullivan et al., 1965; Thorndike and Stein, 1937). This led some scholars to demur that “social intelligence is just general intelligence applied to social situations” (Wechsler, 1958, p. 75).

The search for a social dimension of aptitude was resurrected with Salovey and Mayer’s (1990) competency measure of emotional intelligence, which introduced pictorial rather than verbal test items, found to be psychometrically distinct from general intelligence (Mayer, 2014a). It predicts job performance above and beyond general intelligence, albeit primarily in emotion-laden roles such as counseling or human resources (Côté, 2014). Other emotional intelligence researchers introduced self-report inventories rather than competency tests (Bar-On, 2004; Goleman, 2006; Goleman et al., 2013). These correlate more highly with leadership performance, but they overlap so much with measures of personality and of leadership behavior itself that they do not purely measure social intelligence (Mayer et al., 2008). More recent work has argued that a better measurement of emotional intelligence might focus on behaviors rather than just abilities. Such measures could potentially demonstrate predictive validity beyond that offered by general intelligence or personality (Boyatzis, 2018).

Another approach conceptualizing intelligence uses hierarchical models that posit domain-specific abilities underneath the general ability umbrella (Flanagan et al., 2013; MacCann et al., 2014). Studies of academic performance reveal dimensions of math and technical ability distinct from social and verbal ability (Mayer, 2014a; Mayer, 2014b; Wong et al., 1995). Mayer and Skimmyhorn (2017) identified “thing-focused” (spatial and mathematical) and “people-focused” (verbal and personality) abilities among military cadets. People-focused intelligence predicted performance in classes that dealt with more social matters and in supervisor ratings of a cadet’s extra-curricular responsibilities.

Gardner (1983) made a case for multiple intelligences based on evidence about selective impairment by injuries or developmental disorders. Baron-Cohen (2002) explained autism as an extreme thing-orientation rather than people-orientation. An assessment of people-focused ability is the Reading the Eyes in the Mind Test (Baron-Cohen et al., 2005; Baron-Cohen et al., 2001), which correlates with verbal aptitude (Golan et al., 2006). Recent neuroscience research has added new evidence that distinct mental systems are involved in social tasks like recognizing faces and emotional expressions as opposed to abstract cognitive tasks such as math problems (Baron-Cohen et al., 1999; Goleman and Boyatzis, 2008; Kreifelts et al., 2010; Meyer et al., 2015; Smith et al., 2020).

As concepts of intelligence have evolved, so has applied research on how to measure it. The SAT began in 1926 by adapting the U.S. Army’s assessment of mental ability and later split into verbal and quantitative sections. Most research on its predictive validity uses the combined score to predict overall GPA in the first year of college, but some studies analyzed verbal and quantitative scores separately, finding them most predictive of grades in English and mathematics, respectively (Mattern et al., 2012).

The Graduate Management Admissions Test (GMAT) began in 1956 with parallel verbal and quantitative sections consisting of business-focused questions (henceforth, GMAT-V and GMAT-Q). There is evidence that combined scores predict first-year MBA GPA above and beyond the predictiveness of undergraduate GPA (Kuncel et al., 2007). Another section was added to the GMAT in 1994 to assess analytic writing proficiency (GMAT-W) but there has been no substantial external research about its incremental validity (Sireci and Talento-Miller, 2006).

## 2 Hypotheses

### 2.1 Hypotheses on grades

Our initial hypothesis about MBA academic success follows straightforwardly from the two dimensions of talent:

*Hypothesis 1: MBA grades vary on two dimensions, quantitative success, and social and verbal success.*

Next, we consider the *predictors* of performance. Prior literature shows the most reliable predictor of MBA grades is undergraduate grades (Ahmadi et al., 1997; Yang and Lu, 2001), usually overall GPA (Busato et al., 2000; Furnham and Mitchell, 1991; Mellanby et al., 2000; Schunk et al., 2008). However, universities and majors differ in standards, so GPAs are not easily comparable across different contexts. This limitation is one reason why standardized aptitude tests can help predict MBA student academic performance (Carver and King, 1994; Hoefer and Gould, 2000; Kuncel et al., 2007; Oh et al., 2008; Pesta and Poznanski, 2009; Rothstein et al., 1994; Yang and Lu, 2001; Youngblood and Martin, 1982). One meta-analysis found that the combination of quantitative (GMAT-Q) and verbal (GMAT-V) scores account for about 20% of the variance in overall academic performance in MBA programs (Kuncel et al., 2007). However, other scholars argue that combined GMAT does not add predictive utility once undergraduate grades and past work experience are taken into account (Pratt, 2015) or that undergraduate grades more strongly predict first-year GPA compared to GMATs and GREs (Fairfield-Sonn et al., 2010; Li and Cohen, 2024). Other research questions the validity of the GMAT when used for selection entirely (Kass et al., 2019).

Overall, past research has produced mixed findings, and the methodological shortcomings of the studies raise concerns about the generalizability of their conclusions. Chief among these concerns is that performance has typically been assessed through a unidimensional success outcome measure. When performance was examined in different contexts, a meta-analysis documented stronger predictiveness of emotional and social competencies for performance in humanities than sciences (MacCann et al., 2020). Consistent with Hypothesis 1, we argue that success can be along separate social or quantitative dimensions and can be differentially predicted with distinct measures that are available to officers during admission time (i.e., GMAT social scores and GMAT quantitative scores):

*Hypothesis 2a: GMAT-Q positively predicts MBA grades in quantitative subjects.*

*Hypothesis 2b: GMAT-V positively predicts MBA grades in social subjects.*

### 2.2 Social aptitude measures beyond grades

Social intelligence may be measurable through grades in social related classes, but it can also be measured through students’ social interactions and corresponding leadership attainment. One objective measure of leadership attainment comes from the number of positions that a student holds in extracurricular MBA clubs or organizations. These positions are highly sought after in part because students believe that they will be viewed favorably by job recruiters as evidence of leadership capabilities. Students commonly include such roles on their C.V. This documentation of leadership positions in clubs is especially important given that in many top schools GPAs cannot be shared with recruiters.

Another way to measure leadership is through peer perceptions. Broadly construed, leadership involves not just performance in executive positions but all the ways that a person affects others and helps or hinders their work. Most MBA programs, like corporations, involve a great deal of team projects and use peer assessments to give students feedback about their leadership performance on many different subdimensions.

Although past authors have asserted that GMATs predict competencies needed for leadership (e.g., Kuncel et al., 2007), there is little direct evidence. A study at a competitive MBA program found that combined GMATs *positively* predicted GPA but *negatively* predicted measures of leadership, positions held, and peer perceptions (DeRue, 2009). The only positive predictor of leadership performance was a measure of the diversity of past work experiences.

A study that examined verbal and quantitative aptitude tests separately found evidence that the verbal aptitude predicted leadership performance in a one-day simulation. Kass et al. (2012) used a day-long exercise to measure MBA performance in five areas: leadership initiative, decision making, organizing, communication and teamwork. Undergraduate GPA and GMAT-Q predicted none of these competencies, however, verbal aptitudes did: GMAT-V substantially predicted organizing and GMAT-W predicted the other four. Of course, simulations of leadership are more verbally loaded than the real thing, but these results suggest that socio-verbal-related aptitudes predict social performance in the form of leadership prowess. These findings, combined with previous work that has linked “people” oriented interests and social performance, lead us to hypothesize that verbal aptitude predicts leadership measures.

*Hypothesis 3: Verbal aptitude positively predicts objective leadership attainment.*

*Hypothesis 4: Verbal aptitude positively predicts peer-perceived leadership performance.*

### 2.3 Interests and aptitudes as predictors

In research on mental abilities, Cattell (1987) first theorized that interests complement abilities to determine performance. Social intelligence researchers have found that measures of interest in people correlate with later measures of aptitude. For example, the time infants spend looking at faces predicts later size of vocabulary (Lutchmaya et al., 2001, 2002). Childhood interest in people-versus-things predicts later-in-life aptitudes and occupational choices (Ackerman, 2014; Graziano et al., 2012; Rolfhus and Ackerman, 1999). Furthermore, people-versus-things is an overarching factor in the many specific dimensions assessed in vocational interest inventories (Holland, 1966; Tay et al., 2011).

Why are interests important? They predict motivation and the amount of effort a student will exert in a particular area of study. They drive the desire to learn new topics (Silvia, 2008), positively predicting study time, information recall, and academic performance (Silvia, 2006). Consistent with the poet vs. quant distinction reflected in our first hypothesis, we also focus on two broad classes of interests: *people*-focused interests and *thing*-focused interests.

Given the importance of interests in predicting performance, it was necessary that—in addition to expanding our outcome measures in Study 2—we add new predictors of this dimension of interests. Fortunately, almost all admissions offices collect data on interests, whether they know it or not. Applications ask about desired post-MBA industry or domain of work. We refer to this variable as *industry interests*. In addition to industry interests, some MBA programs collect interest-inventory data that reflect *general interests* across thing and people domains. One example tool is *Career Leader* (Butler and Waldroop, 2004). As with industry interests, is also possible to collapse the large variety of general interest responses into the same two categories of thing vs. people-focused interests. We refer to these interests as *general interests.*

Because interests predict academic performance in general, we expect thing-focused general interests amongst MBA students will positively predict quantitative academic performance, and people-focused general interests will positively predict social academic performance and leadership outcomes. We also predict that interests in one domain will negatively predict performance in the opposite domain since students face a resource tradeoff: time and effort spent pursuing interests in one domain detracts from potential time spent investing in the other.

*Hypothesis 5a: Quant interests positively predict GPA-Quantitative.*

*Hypothesis 5b: Poet interests negatively predict GPA-Quantitative.*

*Hypothesis 6a: Quant interests negatively predict GPA-Social.*

*Hypothesis 6b: Poet interests positively predict GPA-Social.*

Furthermore, we anticipate that people-focused interests will predict measures of social performance outside the classroom, including objective leadership performance and peer-rated leadership performance.

*Hypothesis 7a: Quant interests negatively predict objective leadership attainment.*

*Hypothesis 7b: Poet interests positively predict objective leadership attainment.*

*Hypothesis 8a: Quant interests negatively predict peer perceived leadership performance.*

*Hypothesis 8b: Poet interests positively predict peer perceived leadership performance.*

### 2.4 Past activities as predictors

We conducted interviews with MBA admissions staff about the “lore” in their field to understand which experiences recorded in MBA applications impact their admissions decisions. Two prominent pre-application experiences were frequently cited: military experience and participation in college varsity athletics. Practitioners indicated a belief that these experiences signal leadership or teamwork capabilities that would positively impact performance in the MBA program. Due to the unique availability of these data and its use by practitioners in the real world, we wanted to test whether these prior experiences actually predict leadership performance.

*Hypothesis 9a: Varsity experience positively predicts GPA-Social.*

*Hypothesis 9b: Varsity experience positively predicts objective leadership performance.*

*Hypothesis 9c: Varsity experience positively predicts peer perceived leadership performance.*

*Hypothesis 10a: Military experience positively predicts GPA-Social.*

*Hypothesis 10b: Military experience positively predicts objective leadership performance.*

*Hypothesis 10c: Military experience positively predicts peer perceived leadership performance.*

The above hypotheses examine predictors that best forecast our proposed two dimensions, challenging a unidimensional perspective of MBA success. We test these ideas across two studies conducted at a highly selective U.S. MBA program. The research employs a quantitative comparative design using existing institutional data and survey measures, consistent with recommendations for studies that analyze associations between selected predictors and outcomes (Hair et al., 2018; Shadish et al., 2002). Although we collected the archival and survey data from multiple sources, the study remains fully quantitative because the measures were numeric and standardized, and we analyzed them using statistical modeling. Study 1 employs survey data to focus on academic outcomes using course grades and admissions-time aptitude metrics. Study 2, which relies on archival data collected by the school, expands this investigation to further include leadership outcomes and interest-based predictors.

## 3 Study 1

Study 1 explores whether performance falls along two distinct dimensions and if these can be differentially predicted. The study has high ecological validity through using participants from an top-ranked MBA program in the US.^1^ As part of a student-led project, all students from the class of 2014 of a top-10 East Coast MBA program completed a survey about their background and experiences in the school in March 2014 during their final semester. The survey consisted of items measuring the variables listed in section 3.1.2 Study 1 Variables. Specific details regarding response categories appear alongside variable descriptions.

### 3.1 Study 1 method

#### 3.1.1 Study 1 participants

Of the 729 students in the class of 2014, 518 (71.0%) completed at least part of the survey. We excluded participants missing data for any variables in our regression analyses (final N = 322 students). For both this study and Study 2, IRB permission was obtained to request access to archival data from departments in the business school. These departments collected this information as part of their normal student onboarding, instruction, or employment support. Such permission was granted on the condition of strict anonymity of students and protection of the school’s confidential and proprietary data. The respective departments and school offices consented to sharing relevant data under the condition that it is not shared with others. The sample size of the dataset for Study 1 was limited by the number of students in the class; we include all subjects for whom we have full data and excluded those for whom we did not have complete data for variables used in our models. Data were analyzed using R version R 3.6.2 and SPSS.

#### 3.1.2 Study 1 variables

##### 3.1.2.1 Race/ethnicity

Participants selected from one of several options to describe their race. We created dummy indicators (1 if a member of the indicated group, 0 if not) representing the most frequently indicated races: Asian (*N* = 95), African American or Black (*N* = 11), and other (*N* = 11). Subjects who selected White (*N* = 205) were coded as 0 on each of the above. Students selecting multiple races were coded as other.

##### 3.1.2.2 Latinx

Independent of race variables, we included a code for whether students identified as Latinx (1 = yes, 0 = no; *N* = 29).

##### 3.1.2.3 Age

Student self-provided age in years (mean = 28.73, SD = 1.95, minimum = 24, maximum = 36).

##### 3.1.2.4 Gender

Students indicated whether they identified as men (gender = 0; *N* = 209) or women (gender = 1; *N* = 113).

##### 3.1.2.5 U.S. citizenship

Students indicated U.S. citizenship (citizenship = 1; *N* = 205).

##### 3.1.2.6 GMAT-Q and GMAT-V

Students reported GMAT scores both quantitative (GMAT-Q; mean = 46.86, SD = 3.55, min = 30, max = 59) and verbal (GMAT-V; mean = 42.10, SD = 4.01, min = 32, max = 60). The maximum possible score for each section is 60.

##### 3.1.2.7 Undergraduate major

Students provided their undergraduate majors, and we assigned each to three broad domains: business, STEM (science, technology, engineering, mathematics), and other. We created dummy variables for both STEM and business majors (coded 1 or 0). One hundred eleven students were business majors, 87 were STEM, and 124 were other.

##### 3.1.2.8 Quantitative and social GPA in core class grades

Students completed 11 required (“core”) courses. The quantitative courses were: Macroeconomics, Microeconomics, Accounting I, Accounting II, Statistics, Decision Modeling, Corporate Finance, and Operations. These classes are more quantitative in nature and require a relatively heavier emphasis on numbers, formulas, and calculations than other courses. The core social courses were: Marketing I, Corporate Strategy, Change Management, and Organizational Behavior. These classes have a relatively stronger emphasis on people, social behavior, and communication. An additional course, Marketing II, required use of both quantitative and social skills, and thus we did not classify it with either factor. Grades in these are assigned on a five-point scale, labeled as follows: Honors (H), High Pass (HP), Pass (P), Low Pass (LP), and Failure (F), with no plus or minus grades. Participants self-reported grades in each of these classes, and for grade-point average (GPA) calculation, we converted grades to numeric scores from 4 (H) down to 0 (F).

Our first hypothesis proposes that MBA GPA will fall into two dimensions: social and quantitative. As discussed in Results, confirmatory factor analysis (CFA) provides support for this hypothesis. We thus created composite scales to measure quantitative course GPA (GPA-Q; mean = 3.31, SD = 0.41, min = 2.29, max = 4.00) and social course GPA (GPA-S; mean = 3.28, SD = 0.43, min = 2.00, max = 4.00) for use as outcome variables in our later regression analyses. Each scale ranged from 0 to 4.

### 3.2 Study 1 results

Our data analyses employed both confirmatory factor analysis (CFA) and multiple regression. Methodologists have documented that CFA is appropriate for testing multidimensional structures based on theory (Kline, 2023) and multiple regressions are suitable for analyses involving assessing predictive ability among multiple potentially correlated predictors (Aiken and West, 1991). Our first hypothesis was that student grades reflect distinct factors of quantitative and social performance. We expected that classes emphasizing quantitative content associate as one factor and classes emphasizing social interactions of various kinds to load onto a second factor. We tested this prediction with a CFA on grades. A two-factor model [*χ*^2^ = 29.68, *df* = 34, *p* = 0.68, comparative fit index (CFI) = 1.00, Tucker-Lewis index (TLI) = 1.00, root-mean-square error of approximation (RMSEA) = 0.00, and standardized root-mean-square residual (SRMR) = 0.03] fit the data significantly better [*χ*^2^ = 19.38, *df* = 1, *p* < 0.001] than a single-factor model [*χ*^2^ = 49.06, *df* = 35, *p* = 0.06, comparative fit index (CFI) = 0.98, Tucker-Lewis index (TLI) = 0.97, root-mean-square error of approximation (RMSEA) = 0.04, and standardized root-mean-square residual (SRMR) = 0.04]. The data better supported Hypothesis 1, suggesting that academic performance consists of two distinct factors (GPA-Q and GPA-S).

The correlations between two dimensions of MBA performance and the admissions-time predictor variables are shown in Table 1.

**Table 1:** Study 1 means, standard deviations, and correlations.

Variable	*M*	SD	1	2	3	4	5	6	7	8	9	10	11	12	13
1. GPA quant	3.31	0.41													
2. GPA social	3.28	0.43	0.42^**^												
3. African American	0.03	0.18	−0.14^*^	−0.11^*^											
4. Asian	0.30	0.46	0.01	−0.18^**^	−0.12^*^										
5. Race other	0.03	0.18	−0.05	−0.02	−0.04	−0.12^*^									
6. Hispanic	0.09	0.29	−0.09	−0.04	−0.06	−0.20^**^	0.00								
7. US citizen	0.64	0.48	−0.16^**^	0.08	0.11	−0.29^**^	0.04	−0.17^**^							
8. Age	28.73	1.95	−0.21^**^	−0.20^**^	0.04	0.12^*^	−0.03	0.12^*^	−0.12^*^						
9. Woman	0.35	0.48	−0.26^**^	−0.04	0.01	0.04	0.08	−0.07	0.12^*^	−0.09					
10. Undergrad major bus	0.34	0.48	0.02	0.01	0.04	−0.04	0.08	0.00	0.05	−0.11^*^	−0.08				
11. Undergrad major STEM	0.27	0.44	0.12^*^	0.00	0.04	0.14^*^	−0.04	0.10	−0.31^**^	0.19^**^	−0.07	−0.44^**^			
12. Undergrad major other	0.39	0.49	−0.14^*^	−0.01	−0.08	−0.09	−0.04	−0.09	0.24^**^	−0.06	0.14^*^	−0.57^**^	−0.48^**^		
13. GMAT quant	46.86	3.55	0.30^**^	0.03	−0.06	0.20^**^	−0.05	−0.05	−0.17^**^	−0.02	−0.19^**^	−0.10	0.21^**^	−0.10	
14. GMAT verbal	42.09	4.01	0.27^**^	0.23^**^	−0.07	−0.10	0.00	−0.03	0.23^**^	−0.16^**^	−0.11	−0.02	−0.08	0.10	−0.04

M and SD are used to represent mean and standard deviation, respectively. ^*^ Indicates *p* < 0.05. ^**^ Indicates *p* < 0.01.

Hypotheses 2a and 2b predicted that performance in quantitative courses (GPA-Q) follows from quantitative aptitudes, whereas performance in social classes (GPA-S) follows from verbal aptitudes. To test these, we constructed multiple linear regressions with GPA-Q and GPA-S as outcome variables, and GMAT-Q and GMAT-V as predictors (See Table 2, models 2 and 4). We use a common set of controls for regression analyses reported below and, unless otherwise noted, values do not meaningfully differ for models without controls.

**Table 2:** Linear regression of quantitative and social grades on GMAT Q and GMAT V.

Variable	Model 1	Model 2	Model 3	Model 4
	DV = Quantitative course GPA		DV = Social course GPA	
(Intercept)	4.95 (0.32)^***^	2.19 (0.51)^***^	4.42 (0.36)^***^	3.12 (0.60)^***^
African American	−0.31 (0.12)^**^	−0.25 (0.11)*	−0.34 (0.13)^**^	−0.30 (0.13)^*^
Asian	−0.05 (0.05)	−0.08 (0.05)	−0.19 (0.06)^***^	−0.19 (0.06)^***^
Race other	−0.10 (0.12)	−0.10 (0.11)	−0.12 (0.13)	−0.12 (0.13)
LatinX	−0.20 (0.08)^*^	−0.19 (0.07)*	−0.12 (0.09)	−0.12 (0.09)
US Citizen	−0.12 (0.05)^*^	−0.16 (0.05)***	0.04 (0.05)	0.01 (0.06)
Age	−0.05 (0.01)^***^	−0.04 (0.01)***	−0.04 (0.01)^**^	−0.03 (0.01)^*^
Woman	−0.22 (0.04)^***^	−0.15 (0.04)***	−0.04 (0.05)	−0.00 (0.05)
Undergrad major bus	0.05 (0.05)	0.08 (0.05)	0.03 (0.06)	0.04 (0.05)
Undergrad Major STEM	0.15 (0.06)^**^	0.12 (0.05)^*^	0.10 (0.06)	0.09 (0.06)
GMAT quant		0.03 (0.01)^***^		0.01 (0.01)
GMAT verbal		0.03 (0.01)^***^		0.02 (0.01)^**^
*R* ^2^	0.20	0.30	0.10	0.13
Adj. *R*^2^	0.17	0.28	0.07	0.10
RMSE	0.37	0.35	0.42	0.41

Number of observations = 322. ^***^p < 0.001, ^**^p < 0.01, ^*^p < 0.05. Regression coefficients are unstandardized, and standard errors are in parentheses.

We found support for Hypothesis 2a, that quantitative-course GPA was predicted by GMAT-Q (*b* = 0.03, SE = 0.01, *p* < 0.001). Contrary to predictions, it was also predicted by GMAT-V (*b* = 0.03, SE = 0.01, *p* < 0.001). In support of hypothesis 2b, social-course GPA was predicted by GMAT-V (*b* = 0.02, SE = 0.01, *p* = 0.001), but social grades were not predicted by GMAT-Q (*b* = 0.01, SE = 0.01, *p* = 0.27).

### 3.3 Study 1 discussion

Study 1 supported our first hypothesis that MBA performance falls on two dimensions: quantitative and social performance. In addition, supporting Hypotheses 2a and 2b, Study 1 demonstrated that GMAT scores in quantitative or verbal domains predict academic success in those domains. It is worth nothing that verbal aptitude predicted quantitative performance as well. This may reflect that even quantitative problems require verbal expression. Performance in quantitative classes did not predict performance in verbal ones. This supports the vernacular understanding in the MBA community that one kind of talent does not always contribute to the other.

Study 1 shared some common limitations with past research in this area: it relied on self-reported grades and aptitude measures. This is problematic because self-report scores can be distorted by conscious and unconscious processes like misrepresentation (Barrick and Mount, 1996), motivated memory, and retrospective self-deception processes (Dunning et al., 2004; Hollander, 2009). Study 2 expands on Study 1 while addressing these self-report limitations by sourcing grades, test scores, and other admissions-time data directly from students’ educational records. This method also allowed us to broaden the scope of performance measures and predictor measures beyond what is feasible to assess via survey.

## 4 Study 2

Study 2 builds upon Study 1 in three important ways. First, we replicate the findings from Study 1, that academic performance is best captured by two separate dimensions, and that different aptitudes predict performance in those dimensions. Second, we test predictions related to additional predictors of social performance and their validity. Third, we incorporate additional measures of leadership other than grades.

### 4.1 Study 2 method

#### 4.1.1 Study 2 data sources

Data were obtained from the admissions, student life, and career services office, as well as the orientation program of an Ivy League MBA program. As with Study 1, IRB approval was obtained to request access to archival data from departments in the business school. Such access was granted on the condition of strict anonymity and protection of the data. The respective departments and school offices consented to sharing relevant data under the condition that it is not shared outside of the author team. The nature of the archival dataset is described in further detail within sections 4.1.3 Study 2 Predictors, 4.1.4 Study 2 Outcomes, and 4.1.5 Study 2 Controls. Distinct data sets sourced from various parts of the school were combined using student names as the key variable. Archival academic, admissions, and leadership records are commonly used in higher-education performance research because they reduce self-report bias and provide objective behavioral outcomes (Galla et al., 2019; Robbins et al., 2004). Sample sizes of datasets for both Study 1 and Study 2 were limited by the number of students in each class; we include all subjects for whom we have full data and excluded those for whom we did not have complete data for variables used in our models. Data were analyzed using R version R 3.6.2 and SPSS.

#### 4.1.2 Study 2 participants

We collected data on 1,324 graduates from the classes of 2009 and 2010. We excluded participants missing data for any variables used in our full regression models, resulting in 698 subjects (Mean age = 27.50, SD = 2.26, median = 27.21, minimum = 21.7, maximum = 38.4). Most participants were male (65.2%) and US citizens (73.50%). On average, participants had 4.90 years of full-time work experience (SD = 1.9, median = 4.7, minimum = 0, maximum = 12.3), though we do not include this in our analyses as it is highly correlated with age, which we do include. Participants held jobs in a diverse set of industries including financial services (*n* = 223, 31.9%), consulting (*n* = 111, 15.9%), and technology (n = 76, 10.9%).

#### 4.1.3 Study 2 predictors

##### 4.1.3.1 GMAT

In the years 2009 and 2010, there were 3 GMAT sections: analytical writing (GMAT-W), quantitative (GMAT-Q) and verbal (GMAT-V).^2^ Scores can range from 0 to 60 for the verbal and quantitative sections, and 1–6 on the writing section. In our sample, the mean GMAT-Q was 46.48 (SD = 3.27, minimum = 33, maximum = 51), the mean GMAT-V was 40.75 (SD = 3.86, minimum = 25, maximum = 51), and the mean GMAT-W score was 5.22 (SD = 0.69, minimum = 3, maximum = 6).

##### 4.1.3.2 Poet/Quant general interests

Students completed a popular vocational interest inventory,^3^ which presents approximately 190 different work activities (e.g., design a scientific experiment, coach a sports team). They indicated interest in each item on a four-point scale. This range of items is fairly broad and should fall on two basic factors corresponding to Baron-Cohen’s (2003) dimensions of systematizing and empathizing. Therefore, we conducted a principal components analysis (PCA) to assign each of the Career Leader items to one of these two dimensions. Including data from all students who completed the inventory, we ran a PCA with varimax rotation, specifying two principal components solution. The first of the components accounted for 12.9% of the total post rotation variance in the items, and the second component 10.9%.

Based on the above, we created composite scales using the items that loaded the highest on each component. Each of the scales had high internal consistency: the first scale represented the dimension of *poet* interests (e.g., “train business professionals in team dynamics;” “marketing brand manager,” “lead a company task force studying personnel policy,” “develop an advertising campaign for a product,” “public relations professional,” and “counsel families in crisis”; Cronbach’s alpha = 0.94). The second scale represented a dimension of *quant* interests (e.g., “create a computer model for analyzing financial markets,” “financial analyst,” “mathematician,” “create a complex airline route plan,” “use mathematical modeling to study weather systems,” and “manage a portfolio of stocks for an investment company” (Cronbach’s alpha = 0.93). In our sample, the mean poet general interest score was 1.37 (SD = 0.52, minimum = 0, maximum = 2.76) and the mean quant general interest score was 1.25 (SD = 0.52, minimum = 0.31, maximum = 2.91).

##### 4.1.3.3 Poet/quant industry interests

Students indicated their desired industry interests, and we categorized responses into three exclusive categories: finance (*n* = 268), management (*n* = 129), and other (*n* = 301). A career interest in finance is our proxy for quant interests and a career interest in management is our proxy for poet interests. We included binary variables for both management and finance in our models (1 = interest, 0 = no interest).

##### 4.1.3.4 Military experience

We include a binary variable indicating whether students indicated prior military service (in any country) on their admissions applications (1 = yes, 0 = no). 23 (3.3%) students had military experience.

##### 4.1.3.5 Varsity experience

Students indicated undergraduate extracurricular activities, including participation in a varsity sport. Students with varsity experience were coded as 1 (38 students, or 5.4%), those with no experience are coded as 0.

#### 4.1.4 Study 2 outcomes

##### 4.1.4.1 Quantitative GPA (GPA-Q) and social GPA (GPA-S)

Grades were obtained from students’ academic records. We used the same process as in Study 1 to make a distinction between courses heavy in quantitative content and those that emphasize non-quantitative skills (“soft skills”). As in Study 1, grades are assigned on a five-point scale, although the specific grade points awarded differed from the Study 1 dataset. The grades and corresponding points in this dataset were: Honors (H) = 10, High Pass (HP) = 7, Pass (P) = 4, Low Pass (LP) = 1, and Failure (F) = 0, with no plus or minus grades awarded. As in Study 1, we conducted a CFA to test a model whether the courses were representative of common underlying conceptual factors. Based on results of this analysis (highlighted in Results section), we confirmed that grades were best represented by the same Poet and Quant factors as found in Study 1. We averaged grades for the quantitative and social subject courses to create GPA-Q and GPA-S scales (1–10) and multiplied by 0.4 to reach a four-point scale (GPA-Q mean = 2.93, SD = 0.58, minimum = 1.43, maximum = 4.0; GPA-S mean = 2.90, SD = 0.55, minimum = 1.2, maximum = 4.0).

##### 4.1.4.2 Objective leadership performance—club leadership

MBA students often participate in extracurricular clubs during their MBA (e.g., investment banking club, marketing association, women in business, Canadian students’ club, wine appreciation, snow sports club, student government roles). They demonstrate objective leadership by serving as an ‘officer’ or other leadership role within them. We operationalized objective leadership performance through measuring each student’s number of club leadership positions (mean = 1.25, SD = 1.27, minimum = 0, maximum = 6).

##### 4.1.4.3 Perceived leadership performance—peer perceptions of assertiveness and inclusiveness

As part of the required leadership course, students solicited feedback from classmates on a list of different leadership-relevant items. These data provide insight on how students were perceived by peers. Classmates’ responses to each item were measured using a 7-point scale, with higher scores indicating agreement that the item’s content was true of the target student.

From the dozens of items on the feedback form, we identified two groupings of questions that were relevant for perceived leadership by classmates. From these we created two composite scales measuring two dimensions of peer-perceived leadership for each student: assertiveness (Cronbach’s alpha 0.85) and inclusiveness (Cronbach’s alpha = 0.88). We considered these dimensions relevant, as assertiveness involves sharing an individual’s preferred opinion or directives to others, and inclusiveness involves requesting opinions and agreeing upon direction from the social collective. The questions that comprised the assertiveness scale are “speaks up and shares own views when appropriate,” “able to stand own ground in a heated conflict,” “willing to engage in constructive interpersonal confrontations,” “able to use vivid images and compelling logic and facts to support argument,” and “willing to engage in constructive interpersonal confrontations.” The questions that comprised the inclusiveness scale are “competitive side comes out to an excessive extent” (reverse coded), “relentless and pushy in own requests of others” (reverse coded), “listens effectively to criticism or alt point of view,” “when someone else is speaking, interrupts or shows impatience” (reverse coded), and “flexible and tries to accommodate others’ needs.” Mean assertiveness was 5.31 (SD = 0.68, min = 2.25, max = 6.75) and mean inclusiveness was 5.71 (SD = 0.65, min = 2.50, max = 7.00).

#### 4.1.5 Study 2 controls

##### 4.1.5.1 Undergraduate grade point average

Undergraduate GPA ranges from 0.0 to 4.0 for most universities in the United States (GPAs from non- US schools are converted to the same four-point scale). Some researchers have excluded non-US students from models like GPA because of the difficulty in converting other grading systems to their US equivalent (e.g., McClure et al., 1986). The mean undergraduate GPA in our sample was 3.42 (SD = 0.38, minimum = 1.53, maximum = 4.00).

##### 4.1.5.2 Undergraduate major

Majors provided by students in their admissions applications were divided into three broad domains: business, STEM (science, technology, engineering, mathematics), and other. We used dummy variable for both STEM and business majors (rated 1 or 0). Three hundred thirty-one students were business majors, 191 were STEM, and 176 were other.

##### 4.1.5.3 Citizenship status

We treated citizenship as a dichotomous variable, with non-US citizens coded as 0 and US citizens coded as 1. Five hundred thirteen students were US citizens.

##### 4.1.5.4 Gender

Gender was collected by the school as a dichotomous variable, with men coded as 0 and women coded as 1. 34.8% of the students were female.

##### 4.1.5.5 Financial aid status

We coded whether students received any financial aid (1 = yes, 0 = no). One hundred sixty-three students received financial aid.

##### 4.1.5.6 Age

We calculated age from the date on which data analysis commenced. Mean age for our sample was 27.5 (SD = 2.25, minimum = 21.67, maximum = 38.42).

##### 4.1.5.7 Ethnicity

We created an indicator variable for each of the largest ethnic groups in the sample: African-American or Black (*N* = 36), East Asian (*N* = 125), Latinx (*N* = 40), South Asian (*N* = 55), and a category for other non-White groups (*n* = 41). We coded these indicators as 0 if a student was not a member of the ethnic group and 1 if they were part of the group. Zeroes on all indicators indicated a White, non-Latinx student.

### 4.2 Study 2 results

Again, we employed both confirmatory factor analysis (CFA) and multiple regression. We documented in the Study 1 Results section that such analyses are well-justified (see Aiken and West, 1991; Kline, 2023). We first replicated our findings from Study 1, supporting Hypothesis 1, that grades split along social and quantitative dimensions. We conducted a CFA on grades and again found evidence that a two-factor model [χ^2^ = 98.81, df = 53, comparative fit index (CFI) = 0.98, Tucker-Lewis index (TLI) = 0.97, root-mean-square error of approximation (RMSEA) = 0.03, and standardized root-mean-square residual (SRMR) = 0.04] fit the data better than a single-factor model [χ^2^ = 178.61, df = 54, comparative fit index (CFI) = 0.94, Tucker-Lewis index (TLI) = 0.93, root-mean-square error of approximation (RMSEA) = 0.06, and standardized root-mean-square residual (SRMR) = 0.05; for the difference between these two nested models: χ2 = 79.80, df = 1, *p* < 0.001]. Therefore, the data in Study 2 again supported separating academic outcomes into two factors, GPA-Q and GPA-S.

The remainder of our hypotheses pertained to relationships between student academic and leadership outcomes and various aptitude, interests, and experience predictors. We enter a common set of controls for regression analyses and unless otherwise noted, the effects are not meaningfully different for a model without controls. The controls are undergraduate GPA, US citizenship, gender, financial aid, age, ethnicity, and undergraduate major (STEM, Business, or other). Correlations between all DVs, IVs, and controls are presented in Table 3.

**Table 3:** Correlations between Study 2 variables (predictors and outcomes).

Variable	*M*	SD	1	2	3	4	5	6	7	8	9	10	11	12	13	14	15	16	17	18	19	20	21	22	23	24	25	26
1. GPA quant	2.92	0.58																										
2. GPA social	2.90	0.55	0.43^**^																									
3. Club positions	1.25	1.27	−0.06	0.10^**^																								
4. Inclusive	5.71	0.65	−0.13^**^	−0.12^**^	−0.01																							
5. Assertive	5.31	0.68	0.24^**^	0.23^**^	−0.01	−0.10^**^																						
6. African American	0.05	0.22	−0.27^**^	−0.12^**^	−0.02	0.05	−0.04																					
7. Asian	0.18	0.38	−0.04	−0.27^**^	−0.11^**^	0.13^**^	−0.26^**^	−0.11^**^																				
8. LatinX	0.10	0.30	0.04	0.05	0.04	−0.04	0.01	−0.08^*^	−0.15^**^																			
9. Race other	0.08	0.27	−0.01	0.04	0.10^*^	0.05	−0.05	−0.07	−0.14^**^	−0.10^*^																		
10. SE Asian	0.06	0.23	0.00	−0.07	0.01	0.01	0.03	−0.06	−0.12^**^	−0.08^*^	−0.07																	
11. US Citizen	0.73	0.44	−0.19^**^	0.07	0.12^**^	0.02	0.04	0.11^**^	−0.18^**^	−0.14^**^	0.07	−0.17^**^																
12. Financial Aid	0.23	0.42	−0.18^**^	−0.04	0.10^*^	0.07	−0.06	0.22^**^	−0.07	−0.06	−0.04	−0.06	0.25^**^															
13. Age	27.50	2.26	−0.12^**^	−0.03	−0.07	0.03	−0.00	0.07	0.04	−0.03	−0.01	0.02	−0.09^*^	−0.05														
14. Woman	0.35	0.48	−0.25^**^	−0.05	0.16^**^	0.01	−0.20^**^	−0.02	0.14^**^	−0.04	0.02	−0.02	0.13^**^	0.11^**^	−0.14^**^													
15. Undergrad GPA	3.42	0.38	0.22^**^	0.16^**^	0.00	−0.05	−0.00	−0.17^**^	0.02	0.05	0.01	−0.13^**^	0.09^*^	0.04	−0.18^**^	0.19^**^												
16. Undergrad Major Business	0.47	0.50	0.07	−0.04	−0.07	0.04	−0.04	−0.05	0.07	−0.05	0.02	0.03	−0.01	−0.02	−0.16^**^	−0.03	0.09^*^											
17. Undergrad major STEM	0.27	0.45	0.18^**^	0.05	−0.03	−0.11^**^	0.03	0.03	−0.05	0.11^**^	0.12^**^	0.03	−0.13^**^	−0.03	0.13^**^	−0.14^**^	−0.09^*^	−0.58^**^										
18. Undergrad major other	0.25	0.43	−0.26^**^	−0.00	0.11^**^	0.07	0.02	0.03	−0.03	−0.06	−0.15^**^	−0.06	0.14^**^	0.05	0.05	0.18^**^	−0.01	−0.55^**^	−0.36^**^									
19. GMAT quant	46.48	3.27	0.38^**^	0.00	−0.08^*^	−0.05	−0.02	−0.19^**^	0.24^**^	−0.02	0.05	0.01	−0.25^**^	−0.16^**^	0.03	−0.18^**^	0.01	−0.01	0.23^**^	−0.23^**^								
20. GMAT verbal	40.74	3.86	0.20^**^	0.27^**^	0.06	−0.11^**^	0.17^**^	−0.18^**^	−0.13^**^	−0.01	−0.02	−0.15^**^	0.18^**^	0.03	−0.03	−0.06	0.05	0.01	−0.09^*^	0.09^*^	−0.12^**^							
21. GMAT writing	5.22	0.69	0.02	0.22^**^	0.15^**^	−0.08^*^	0.04	−0.05	−0.14^**^	−0.05	−0.02	−0.20^**^	0.29^**^	0.09^*^	−0.18^**^	0.07	0.09^*^	−0.02	−0.13^**^	0.15^**^	−0.10^**^	0.30^**^						
22. General interests quant	1.25	0.52	0.23^**^	−0.01	−0.12^**^	−0.08^*^	0.13^**^	0.02	−0.04	0.02	−0.01	0.08^*^	−0.11^**^	−0.06	0.09^*^	−0.33^**^	−0.08^*^	−0.02	0.21^**^	−0.19^**^	0.21^**^	0.01	−0.15^**^					
23. General interests poet	1.37	0.52	−0.14^**^	0.03	0.15^**^	0.09^*^	0.08^*^	0.06	0.00	−0.01	0.01	0.00	0.09^*^	0.14^**^	−0.00	0.20^**^	0.06	−0.08^*^	−0.11^**^	0.20^**^	−0.14^**^	0.04	0.07	0.22^**^				
24. Industry interests quant	0.38	0.49	0.12^**^	−0.02	−0.12^**^	0.01	0.06	0.04	0.02	−0.03	−0.06	0.03	0.03	−0.08^*^	0.05	−0.13^**^	−0.00	0.19^**^	−0.09^*^	−0.13^**^	0.06	0.04	−0.08^*^	0.25^**^	−0.17^**^			
25. Industry interests poet	0.18	0.39	−0.07	−0.02	0.05	0.02	−0.07	−0.04	0.01	0.01	−0.00	0.01	−0.07	0.04	0.07^*^	−0.01	−0.03	−0.04	0.03	0.01	0.00	0.03	0.04	−0.06	0.07	−0.38^**^		
26. Varsity	0.05	0.23	−0.07	0.01	0.03	0.06	0.05	0.09^*^	−0.05	−0.06	−0.07	−0.06	0.13^**^	0.08^*^	0.02	−0.00	−0.07	−0.04	−0.08^*^	0.12^**^	−0.11^**^	0.03	0.06	−0.01	0.03	−0.03	−0.00	
27. Armed forces	0.03	0.18	−0.04	−0.06	−0.07	0.04	0.03	0.07	−0.09^*^	−0.06	−0.02	0.06	0.02	0.07	0.09^*^	−0.13^**^	−0.06	−0.05	0.08^*^	−0.03	0.00	0.02	0.03	0.07	0.07^*^	0.02	0.02	0.10^*^

M and SD are used to represent mean and standard deviation, respectively. *Indicates *p* < 0.05. **Indicates *p* < 0.01.

#### 4.2.1 Academic predictors

Predictors of performance in quantitative courses are presented in Table 4. Replicating Study 1’s results in support of Hypothesis 2a, GMAT-Q positively predicted GPA-Q (*b* = 0.05, SE = 0.01, *p* < 0.001). GMAT-V also positively predicted GPA-Q (*b* = 0.03, SE = 0.01, *p* < 0.001).

**Table 4:** Study 2 variable effect on MBA quantitative GPA.

Variable	Model 1	Model 2	Model 3	Model 4	Model 5	Model 6	Model 7
(Intercept)	2.66 (0.32)^***^	−1.19 (0.49)^*^	2.57 (0.32)^***^	2.68 (0.32)^***^	2.66 (0.32)^***^	2.65 (0.32)^***^	−1.20 (0.49)^*^
African American	−0.56 (0.09)^***^	−0.31 (0.09)^***^	−0.56 (0.09)^***^	−0.58 (0.09)^***^	−0.56 (0.09)^***^	−0.56 (0.09)^***^	−0.33 (0.09)^***^
Asian	−0.13 (0.05)^*^	−0.17 (0.05)^***^	−0.13 (0.05)^*^	−0.13 (0.05)^*^	−0.13 (0.05)^*^	−0.13 (0.05)^*^	−0.17 (0.05)^**^
Other	−0.13 (0.07)	−0.08 (0.06)	−0.12 (0.07)	−0.13 (0.07)	−0.13 (0.07)	−0.14 (0.07)^*^	−0.08 (0.06)
SE Asian	−0.17 (0.07)^*^	−0.16 (0.07)^*^	−0.15 (0.07)^*^	−0.15 (0.07)^*^	−0.17 (0.07)^*^	−0.17 (0.07)^*^	−0.14 (0.07)^*^
LatinX	−0.12 (0.09)	−0.01 (0.08)	−0.13 (0.08)	−0.12 (0.09)	−0.12 (0.09)	−0.11 (0.09)	−0.00 (0.08)
US citizen	−0.18 (0.05)^***^	−0.16 (0.05)^***^	−0.17 (0.05)^***^	−0.19 (0.05)^***^	−0.18 (0.05)^***^	−0.18 (0.05)^***^	−0.17 (0.05)^***^
Financial aid	−0.13 (0.05)^**^	−0.12 (0.04)^**^	−0.12 (0.05)^*^	−0.12 (0.05)^*^	−0.13 (0.05)^**^	−0.13 (0.05)^**^	−0.10 (0.04)^*^
Age	−0.03 (0.01)^***^	−0.03 (0.01)^***^	−0.03 (0.01)^***^	−0.03 (0.01)^***^	−0.03 (0.01)^***^	−0.03 (0.01)^***^	−0.03 (0.01)^***^
Woman	−0.28 (0.04)^***^	−0.20 (0.04)^***^	−0.21 (0.04)^***^	−0.27 (0.04)^***^	−0.28 (0.04)^***^	−0.29 (0.04)^***^	−0.17 (0.04)^***^
Undergrad GPA	0.36 (0.05)^***^	0.35 (0.05)^***^	0.37 (0.05)^***^	0.36 (0.05)^***^	0.36 (0.05)^***^	0.36 (0.05)^***^	0.35 (0.05)^***^
Undergrad major business	0.21 (0.05)^***^	0.18 (0.05)^***^	0.17 (0.05)^***^	0.19 (0.05)^***^	0.21 (0.05)^***^	0.21 (0.05)^***^	0.16 (0.05)^***^
Undergrad major STEM	0.37 (0.05)^***^	0.29 (0.05)^***^	0.30 (0.06)^***^	0.37 (0.05)^***^	0.37 (0.06)^***^	0.38 (0.05)^***^	0.27 (0.05)^***^
GMAT quant		0.05 (0.01)^***^					0.05 (0.01)^***^
GMAT verbal		0.03 (0.01)^***^					0.03 (0.00)^***^
GMAT written		0.01 (0.03)					0.03 (0.03)
General interest poet			−0.09 (0.04)^*^				−0.05 (0.04)
General interest quant			0.18 (0.04)^***^				0.11 (0.04)^**^
Industry interest poet				−0.06 (0.05)			−0.08 (0.05)
Industry interest quant				0.10 (0.04)^*^			0.03 (0.04)
Varsity					0.00 (0.09)		0.05 (0.08)
Armed forces						−0.17 (0.11)	−0.18 (0.10)
*R* ^2^	0.29	0.39	0.31	0.30	0.29	0.29	0.40
Adj. *R*^2^	0.27	0.37	0.29	0.28	0.27	0.28	0.39
RMSE	0.50	0.46	0.49	0.49	0.50	0.50	0.46

Number of observations = 698. ^***^p < 0.001, ^**^p < 0.01, ^*^p < 0.05.

Predictors of academic performance in social courses are presented in Table 5. As predicted by Hypothesis 2b, GMAT-V strongly predicts GPA-S (*b* = 0.03, SE = 0.01, *p* < 0.001). GMAT-W also predicts GPA-S (*b* = 0.11, SE = 0.03 *p* < 0.001).

**Table 5:** Study 2 variable effects on MBA social GPA.

Variable	Model 1	Model 2	Model 3	Model 4	Model 5	Model 6	Model 7
(Intercept)	2.29 (0.34)^***^	0.04 (0.53)	2.26 (0.34)^***^	2.28 (0.34)^***^	2.28 (0.34)^***^	2.26 (0.34)^***^	−0.15 (0.54)
African American	−0.33 (0.09)^***^	−0.16 (0.10)	−0.34 (0.09)^***^	−0.33 (0.10)^***^	−0.33 (0.09)^***^	−0.33 (0.09)^***^	−0.16 (0.10)
Asian	−0.42 (0.06)^***^	−0.37 (0.06)^***^	−0.43 (0.06)^***^	−0.42 (0.06)^***^	−0.42 (0.06)^***^	−0.43 (0.06)^***^	−0.38 (0.06)^***^
Other	−0.05 (0.07)	−0.01 (0.07)	−0.06 (0.07)	−0.05 (0.07)	−0.05 (0.07)	−0.06 (0.07)	−0.03 (0.07)
SE Asian	−0.06 (0.08)	−0.02 (0.07)	−0.07 (0.08)	−0.06 (0.08)	−0.06 (0.08)	−0.07 (0.08)	−0.03 (0.08)
LatinX	−0.23 (0.09)^**^	−0.09 (0.09)	−0.23 (0.09)^**^	−0.23 (0.09)^**^	−0.23 (0.09)^*^	−0.23 (0.09)^*^	−0.08 (0.09)
US citizen	0.03 (0.05)	−0.02 (0.05)	0.03 (0.05)	0.03 (0.05)	0.03 (0.05)	0.03 (0.05)	−0.03 (0.05)
Financial aid	−0.06 (0.05)	−0.07 (0.05)	−0.07 (0.05)	−0.06 (0.05)	−0.06 (0.05)	−0.06 (0.05)	−0.07 (0.05)
Age	0.00 (0.01)	0.00 (0.01)	−0.00 (0.01)	0.00 (0.01)	−0.00 (0.01)	0.00 (0.01)	0.01 (0.01)
Woman	−0.04 (0.04)	−0.02 (0.04)	−0.07 (0.05)	−0.05 (0.04)	−0.04 (0.04)	−0.05 (0.04)	−0.06 (0.05)
Undergrad GPA	0.21 (0.06)^***^	0.21 (0.05)^***^	0.21 (0.06)^***^	0.21 (0.06)^***^	0.21 (0.06)^***^	0.21 (0.05)^***^	0.20 (0.05)^***^
Undergrad major business	−0.01 (0.05)	0.01 (0.05)	0.01 (0.05)	−0.00 (0.05)	−0.01 (0.05)	−0.01 (0.05)	0.03 (0.05)
Undergrad major STEM	0.06 (0.06)	0.09 (0.06)	0.09 (0.06)	0.06 (0.06)	0.07 (0.06)	0.07 (0.06)	0.13 (0.06)^*^
GMAT quant		0.01 (0.01)					0.01 (0.01)
GMAT verbal		0.03 (0.01)^***^					0.03 (0.01)^***^
GMAT written		0.11 (0.03)^***^					0.11 (0.03)^***^
General interest poet			0.07 (0.04)				0.07 (0.04)
General interest quant			−0.05 (0.04)				−0.05 (0.05)
Industry interest poet				−0.04 (0.06)			−0.06 (0.05)
Industry interest quant				−0.03 (0.05)			−0.01 (0.05)
Varsity					0.04 (0.09)		0.06 (0.09)
Armed forces						−0.22 (0.11)	−0.28 (0.11)^*^
*R* ^2^	0.13	0.19	0.13	0.13	0.13	0.13	0.20
Adj. *R*^2^	0.11	0.17	0.12	0.11	0.11	0.12	0.17
RMSE	0.52	0.51	0.52	0.52	0.52	0.52	0.50

Number of observations = 698. ^***^p < 0.001, ^**^p < 0.01, ^*^p < 0.05.

Hypothesis 3 proposed that verbal aptitude (i.e., GMAT-V and GMAT-W scores) would predict objective leadership performance in student clubs. To test this, we regressed the number of leadership positions held on GMAT scores. Results from this model are highlighted in Table 6. GMAT-W significantly and positively predicted club leadership positions (*b* = 0.20, SE = 0.08, *p* = 0.008) while GMAT-V did not. Neither GMAT-Q nor undergraduate GPA predicted leadership positions significantly.

**Table 6:** Study 2 variable effect on number of club officer positions.

Variable	Model 1	Model 2	Model 3	Model 4	Model 5	Model 6	Model 7
(Intercept)	2.27 (0.81)^**^	0.51 (1.30)	2.20 (0.81)^**^	2.23 (0.81)^**^	2.25 (0.81)^**^	2.24 (0.81)^**^	−0.02 (1.30)
African American	−0.22 (0.23)	−0.11 (0.23)	−0.25 (0.22)	−0.19 (0.23)	−0.23 (0.23)	−0.22 (0.22)	−0.10 (0.23)
Asian	−0.28 (0.13)^*^	−0.21 (0.14)	−0.29 (0.13)^*^	−0.27 (0.13)^*^	−0.27 (0.13)^*^	−0.29 (0.13)^*^	−0.25 (0.14)
Other	0.25 (0.17)	0.29 (0.17)	0.22 (0.17)	0.24 (0.17)	0.25 (0.17)	0.23 (0.17)	0.25 (0.17)
SE Asian	0.46 (0.18)^*^	0.51 (0.18)^**^	0.42 (0.18)^*^	0.44 (0.18)^*^	0.47 (0.18)^*^	0.45 (0.18)^*^	0.44 (0.18)^*^
LatinX	0.17 (0.21)	0.30 (0.22)	0.16 (0.21)	0.18 (0.21)	0.17 (0.21)	0.18 (0.21)	0.32 (0.22)
US citizen	0.19 (0.12)	0.13 (0.12)	0.17 (0.12)	0.22 (0.12)	0.19 (0.12)	0.20 (0.12)	0.13 (0.12)
Financial aid	0.21 (0.12)	0.21 (0.12)	0.17 (0.12)	0.19 (0.12)	0.21 (0.12)	0.23 (0.12)	0.16 (0.12)
Age	−0.02 (0.02)	−0.02 (0.02)	−0.02 (0.02)	−0.02 (0.02)	−0.03 (0.02)	−0.02 (0.02)	−0.01 (0.02)
Woman	0.37 (0.11)^***^	0.38 (0.11)^***^	0.24 (0.11)^*^	0.35 (0.11)^***^	0.37 (0.11)^***^	0.35 (0.11)^***^	0.24 (0.11)^*^
Undergrad GPA	−0.14 (0.13)	−0.14 (0.13)	−0.15 (0.13)	−0.13 (0.13)	−0.13 (0.13)	−0.14 (0.13)	−0.15 (0.13)
Undergrad major business	−0.29 (0.12)^*^	−0.26 (0.12)^*^	−0.19 (0.12)	−0.24 (0.12)^*^	−0.28 (0.12)^*^	−0.28 (0.12)^*^	−0.15 (0.12)
Undergrad major STEM	−0.23 (0.14)	−0.19 (0.14)	−0.08 (0.14)	−0.23 (0.14)	−0.23 (0.14)	−0.22 (0.14)	−0.05 (0.15)
GMAT quant		0.00 (0.02)					0.01 (0.02)
GMAT verbal		0.01 (0.01)					0.01 (0.01)
GMAT written		0.20 (0.08)^**^					0.18 (0.08)^*^
General interest poet			0.33 (0.10)^***^				0.32 (0.10)^**^
General interest quant			−0.27 (0.10)^**^				−0.23 (0.11)^*^
Industry interest poet				0.10 (0.13)			0.08 (0.13)
Industry interest quant				−0.19 (0.11)			−0.08 (0.11)
Varsity					0.10 (0.21)		0.14 (0.21)
Armed forces						−0.39 (0.27)	−0.54 (0.27)^*^
*R* ^2^	0.07	0.08	0.09	0.08	0.07	0.07	0.11
Adj. *R*^2^	0.06	0.06	0.07	0.06	0.05	0.06	0.08
RMSE	1.24	1.23	1.23	1.23	1.24	1.24	1.22

Number of observations = 698. ^***^p < 0.001, ^**^p < 0.01, ^*^p < 0.05.

Hypothesis 4 stated that verbal aptitude would predict peer rated perceptions of leadership. We created separate regression models for our two perceived leadership scales, inclusiveness (Table 7) and assertiveness (Table 8). Inclusiveness was significantly and negatively predicted by GMAT-V (*b* = −0.02, SE = 0.01, *p* = 0.028). Assertiveness was significantly and positively predicted by GMAT-V (*b* = 0.02, SE = 0.01, *p* = 0.001). Overall, there is mixed support for Hypothesis 4, GMAT-V positively predicts assertiveness while negatively predicting inclusiveness. GMAT-W did not significantly predict either domain.

**Table 7:** Variable effect on MBA perceived leadership attainment (Inclusiveness).

Variable	Model 1	Model 2	Model 3	Model 4	Model 5	Model 6	Model 7
(Intercept)	5.71 (0.42)^***^	7.25 (0.68)^***^	5.72 (0.42)^***^	5.72 (0.42)^***^	5.68 (0.42)^***^	5.73 (0.42)^***^	7.18 (0.68)^***^
African American	0.17 (0.12)	0.06 (0.12)	0.16 (0.12)	0.17 (0.12)	0.16 (0.12)	0.16 (0.12)	0.04 (0.12)
Asian	0.30 (0.07)^***^	0.28 (0.07)^***^	0.30 (0.07)^***^	0.30 (0.07)^***^	0.30 (0.07)^***^	0.31 (0.07)^***^	0.27 (0.07)^***^
Other	0.07 (0.09)	0.05 (0.09)	0.06 (0.09)	0.07 (0.09)	0.08 (0.09)	0.08 (0.09)	0.05 (0.09)
SE Asian	0.26 (0.09)^**^	0.24 (0.09)^*^	0.24 (0.09)^*^	0.26 (0.10)^**^	0.26 (0.09)^**^	0.26 (0.09)^**^	0.23 (0.09)^*^
LatinX	0.16 (0.11)	0.08 (0.11)	0.16 (0.11)	0.15 (0.11)	0.16 (0.11)	0.15 (0.11)	0.07 (0.11)
US Citizen	0.04 (0.06)	0.06 (0.06)	0.03 (0.06)	0.04 (0.06)	0.03 (0.06)	0.04 (0.06)	0.05 (0.06)
Financial aid	0.12 (0.06)	0.12 (0.06)^*^	0.10 (0.06)	0.12 (0.06)	0.12 (0.06)	0.11 (0.06)	0.10 (0.06)
Age	0.01 (0.01)	0.01 (0.01)	0.01 (0.01)	0.01 (0.01)	0.01 (0.01)	0.01 (0.01)	0.00 (0.01)
Woman	−0.06 (0.05)	−0.08 (0.06)	−0.11 (0.06)^*^	−0.05 (0.06)	−0.05 (0.05)	−0.05 (0.05)	−0.12 (0.06)^*^
Undergrad GPA	−0.08 (0.07)	−0.07 (0.07)	−0.08 (0.07)	−0.08 (0.07)	−0.07 (0.07)	−0.07 (0.07)	−0.07 (0.07)
Undergrad major business	−0.08 (0.06)	−0.08 (0.06)	−0.04 (0.06)	−0.08 (0.06)	−0.07 (0.06)	−0.08 (0.06)	−0.06 (0.06)
Undergrad major STEM	−0.24 (0.07)^***^	−0.24 (0.07)^**^	−0.17 (0.07)^*^	−0.24 (0.07)^***^	−0.23 (0.07)^**^	−0.24 (0.07)^***^	−0.17 (0.08)^*^
GMAT quant		−0.01 (0.01)					−0.01 (0.01)
GMAT verbal		−0.02 (0.01)^*^					−0.02 (0.01)^*^
GMAT written		−0.06 (0.04)					−0.07 (0.04)
General interest poet			0.12 (0.05)^*^				0.14 (0.05)^*^
General interest quant			−0.13 (0.05)^*^				−0.16 (0.06)^**^
Industry interest poet				0.04 (0.07)			0.06 (0.07)
Industry interest quant				0.03 (0.06)			0.09 (0.06)
Varsity					0.14 (0.11)		0.14 (0.11)
Armed forces						0.17 (0.14)	0.14 (0.14)
*R* ^2^	0.06	0.07	0.07	0.06	0.06	0.06	0.09
Adj. *R*^2^	0.04	0.05	0.05	0.04	0.04	0.04	0.06
RMSE	0.64	0.64	0.64	0.64	0.64	0.64	0.63

Number of observations = 698. ^***^p < 0.001, ^**^p < 0.01, ^*^p < 0.05.

**Table 8:** Variable effect on MBA perceived leadership attainment (Assertiveness).

Variable	Model 1	Model 2	Model 3	Model 4	Model 5	Model 6	Model 7
(Intercept)	5.50 (0.42)^***^	4.70 (0.67)^***^	5.29 (0.42)^***^	5.50 (0.42)^***^	5.48 (0.42)^***^	5.49 (0.42)^***^	4.44 (0.67)^***^
African American	−0.23 (0.12)^*^	−0.15 (0.12)	−0.25 (0.12)^*^	−0.25 (0.12)^*^	−0.24 (0.12)^*^	−0.23 (0.12)^*^	−0.19 (0.12)
Asian	−0.47 (0.07)^***^	−0.45 (0.07)^***^	−0.48 (0.07)^***^	−0.48 (0.07)^***^	−0.47 (0.07)^***^	−0.48 (0.07)^***^	−0.46 (0.07)^***^
Other	−0.03 (0.11)	0.01 (0.11)	−0.05 (0.11)	−0.03 (0.11)	−0.03 (0.11)	−0.03 (0.11)	−0.00 (0.11)
SE Asian	−0.14 (0.09)	−0.13 (0.09)	−0.15 (0.09)	−0.14 (0.09)	−0.14 (0.09)	−0.14 (0.09)	−0.14 (0.09)
LatinX	−0.25 (0.09)^**^	−0.23 (0.09)^*^	−0.26 (0.09)^**^	−0.24 (0.09)^**^	−0.24 (0.09)^*^	−0.25 (0.09)^**^	−0.24 (0.09)^*^
US citizen	0.05 (0.06)	0.03 (0.06)	0.04 (0.06)	0.04 (0.06)	0.04 (0.06)	0.05 (0.06)	0.01 (0.06)
Financial aid	−0.09 (0.06)	−0.10 (0.06)	−0.11 (0.06)	−0.08 (0.06)	−0.10 (0.06)	−0.09 (0.06)	−0.11 (0.06)
Age	−0.00 (0.01)	−0.01 (0.01)	−0.01 (0.01)	−0.00 (0.01)	−0.00 (0.01)	−0.00 (0.01)	−0.01 (0.01)
Woman	−0.25 (0.05)^***^	−0.23 (0.06)^***^	−0.26 (0.06)^***^	−0.25 (0.05)^***^	−0.25 (0.05)^***^	−0.25 (0.05)^***^	−0.25 (0.06)^***^
Undergrad GPA	0.05 (0.07)	0.05 (0.07)	0.04 (0.07)	0.05 (0.07)	0.05 (0.07)	0.05 (0.07)	0.04 (0.07)
Undergrad major business	−0.06 (0.06)	−0.06 (0.06)	−0.03 (0.06)	−0.07 (0.06)	−0.05 (0.06)	−0.06 (0.06)	−0.03 (0.06)
Undergrad major STEM	−0.01 (0.07)	−0.01 (0.07)	0.01 (0.07)	−0.01 (0.07)	−0.01 (0.07)	−0.01 (0.07)	0.04 (0.08)
GMAT quant		0.00 (0.01)					0.00 (0.01)
GMAT verbal		0.02 (0.01)^**^					0.02 (0.01)^**^
GMAT written		−0.04 (0.04)					−0.03 (0.04)
General interest poet			0.15 (0.05)^**^				0.17 (0.05)^**^
General interest quant			0.05 (0.05)				0.01 (0.06)
Industry interest poet				−0.10 (0.07)			−0.11 (0.07)
Industry interest quant				0.03 (0.06)			0.04 (0.06)
Varsity					0.11 (0.11)		0.13 (0.11)
Armed forces						−0.03 (0.14)	−0.10 (0.14)
*R* ^2^	0.12	0.13	0.14	0.13	0.12	0.12	0.16
Adj. *R*^2^	0.11	0.12	0.12	0.11	0.11	0.10	0.13
RMSE	0.64	0.64	0.63	0.64	0.64	0.64	0.63

Number of observations = 698. ^***^p < 0.001, ^**^p < 0.01, ^*^p < 0.05.

#### 4.2.2 Interest predictors

Hypothesis 5a and 5b stated that quantitative interests would positively predict, and poet interests would negatively predict, GPA-Q. First, we analyzed general interests, as shown in Table 4. As predicted, quantitative general interests significantly predicted GPA-Q (*b* = 0.18, SE = 0.04, *p* < 0.001) and poet general interests negatively predicted it (*b* = −0.09, SE = 0.04, *p* = 0.018). Next, we explored models with industry poet and quant interests as predictors. A desire to work in finance positively predicts GPA-Q (*b* = 0.10, SE = 0.04, *p* = 0.018). A desire to work in management, however, does not significantly predict GPA-Q. Overall, we find strong support across both of our interest operationalization for Hypothesis 5a that quant interests predict GPA-Q. We find partial support for Hypothesis 5b as only one of our two poet interests variables negatively predicts GPA-Q.

Next, we examined Hypotheses 6a and 6b, which posited that poet interests would positively predict, and quant interests would negatively predict, GPA-S. However, neither poet nor quant general interests predicted GPA-S. Likewise, neither poet nor quant industry interests predicted GPA-S. Overall, we found no support for Hypotheses 6a and 6b.

Hypothesis 7a suggested that quant interests would negatively predict objective leadership performance, and this was supported in both of our interest operationalizations (see Table 6). Quant industry interests negatively predict objective leadership performance with marginal significance (*b* = −0.19, SE = 0.11, *p* = 0.080). Quant general interests also negatively predict objective leadership performance (*b* = −0.27, SE = 0.10, *p* = 0.010). Hypothesis 7b predicted that poet interests would positively predict objective leadership performance, and results were mixed depending on which operationalization of poet interests was used: Poet general interests predict objective leadership performance (*b* = 0.33, SE = 0.10, *p* < 0.001), but poet career interests do not.

In Hypothesis 8a we predicted that quant interests would negatively predict peer perceived leadership performance. We found that general quant interests significantly negatively predict inclusiveness (See Table 7, *b* = −0.13, SE = 0.05, *p* = 0.015). Industry quant interests did not predict inclusiveness. General quant interests do not significantly predict assertiveness (See Table 8: *b* = 0.05, SE = 0.05, *p* = 0.389), but do without including controls (*b* = 0.15, SE = 0.05, *p* = 0.003). Industry quant interests did not predict assertiveness.

Hypothesis 8b predicted that poet interests would positively predict peer perceived leadership performance. We found that general poet interests did predict inclusiveness (Table 7, *b* = 0.12, SE = 0.05, *p* = 0.02). Poet industry interests did not predict inclusiveness. General poet interests predicted assertiveness (Table 8, *b* = 0.15, SE = 0.05, *p* = 0.004), though not without controls (*b* = 0.06, SE = 0.00, *p* = 0.192). Poet Industry interests did not predict assertiveness. General poet interests did predict inclusiveness (Table 7, *b* = 0.12, SE = 0.05, *p* = 0.020). Poet industry interests did not predict inclusiveness.

#### 4.2.3 Experience predictors

Hypotheses 9a, 9b, and 9c all predicted a relationship between experience in a varsity sport and relevant academic or leadership outcomes. Experience in a varsity sport did not significantly predict social grades, objective leadership performance, peer ratings of, nor peer ratings of assertiveness. Hypotheses 10a, 10b, and 10c made similar predictions about military experiences positively predicting GPA-S, objective leadership performance, and peer perceived leadership performance. In contrast to Hypothesis 10a, military experience *negatively* predicts social grade (*b* = −0.22, SE = 0.11, *p* = 0.053). It did not predict objective leadership performance, peer ratings of inclusiveness, nor peer ratings of assertiveness.

### 4.3 Study 2 discussion

In Study 2, we brought to bear a more comprehensive, multiply sourced dataset to broaden the findings of Study 1 while addressing its self-report limitations. We replicated the major finding that grades consist of two distinct factors, one quantitative and one social. This supports the idea that we can assess two distinct forms of performance: performance in technical content, and performance in social content.

Furthermore, Study 2 replicated Study 1 results regarding admissions-time aptitude predictors. Supporting Hypotheses 2a and 2b, respectively, we demonstrated that quantitative aptitude predicts quantitative performance, and verbal aptitude—as measured by both GMAT-V and GMAT-W—predicts social performance. Interestingly, GMAT-V also predicted quantitative course performance, potentially reflecting the need for students to convey their quantitative knowledge using words—via exams, papers, or class participation.

We also offered hypotheses regarding a different form of social performance: student leadership. We expected that verbal aptitudes would positively predict both club officer positions (Hypothesis 3) and peer ratings (Hypothesis 4). We found support for these hypotheses as well, although the specific pattern changed depending on the outcome measure. GMAT-W, but not GMAT-V, positively predicted objective leadership performance: students with higher writing aptitude aptitudes tended to hold more club officer positions. GMAT-V negatively predicted peer ratings of inclusiveness, but positively predicted ratings of assertiveness.

Turning to our hypotheses involving interest predictors, we found that, consistent with Hypothesis 5a, quant interests—measured generally or in terms of industry—positively predicted quantitative academic performance, and, consistent with Hypothesis 5b, negatively predicted social academic performance. However, we did not find support for Hypotheses 6a and 6b, which posited the opposite pattern, that poet interests would positively predict social academic performance and quant interests would negatively predict social academic performance.

With respect to leadership as a form of social performance, we found that—confirming Hypotheses 7a and 7b—social interests, whether general or industry specific, positively predicted objective leadership performance. One form of quant interests also negatively predicts social performance. There was far less clarity regarding our hypotheses that interests predict subjective, peer-rated leadership performance. General interests positively predicted inclusiveness, supporting Hypothesis 8a and 8b: poet general interests were positively predictive of inclusiveness, and quant general interests were negatively predictive. However, when we explored industry-specific interests, we did not find significant relationships. Likewise, for assertiveness, we found a mixed pattern of results: when control variables were included, poet general interests predicted assertiveness, and quant interests were unrelated to assertiveness. However, without these control variables, quant interests positively predicted assertiveness, and poet interests were not significantly related to assertiveness. As with inclusiveness, industry-specific interests were not related to assertiveness. It is worth noting that general interests were composite measures involving dozens of items, and industry-specific interests were single-item measures. This difference in measurement may have accounted for why general interests had stronger predictive ability.

Finally, varsity and military experience predictors did not significantly predict social performance in either academic or leadership domains. An exception was that military experience marginally *negatively* predicted academic social performance. Thus, Hypotheses 9a, 9a, and 9c, and 10a, 10b, and 10c were not confirmed. This result is particularly relevant for admissions officers who, based on our interviews, view such experiences positively.

In sum, the results from Study 2 reinforced those of Study 1 in terms of the differentiated poet and quant nature of academic performance, and predictive validity of distinct poet or quant aptitude measures on academic performance. Study 2 also provided clarity as to the relationship between aptitudes measures and actual, real world leadership performance. Furthermore, we demonstrated that poet and quant interests were also associated with respective poet and quant academic performance, and also that poet interests specifically predicted objective leadership performance. We found puzzling results for two peer-rated leadership measures: poet interests being positively predictive of inclusiveness, whereas quant interests were negatively predictive; the results for assertiveness were inconclusive. Finally, two experience predictors of note—varsity and military experience—did not predict social outcomes in either academic or actual leadership domains.

## 5 General discussion

The current research makes six key contributions for management education scholars, business school deans, and admissions officers. It combines the two major themes predominating learning and education research—namely, curriculum and outcomes (Rubin and Dierdorff, 2013) to challenge existing approaches to understanding the MBA curricular experience and success within it.

### 5.1 Talent is multidimensional, not unidimensional

First, our findings challenge a large body of past research on MBA student success, which relies on unidimensional academic performance like overall GPA. (e.g., Carver and King, 1994; Christensen et al., 2012; Gupta and Turek, 2015; Naik and Ragothaman, 2004; Pratt, 2015; Sulaiman and Mohezar, 2006; Wilson and Hardgrave, 1995). Results from both datasets show that a two factor (poet and quant) model fits the data better than a single factor model. Poet and quant grades were also not strongly correlated, supporting MBA folklore that classmate strengths are concentrated in one domain or another.

Second, our studies elucidate the relevance of admissions-time student data in predicting student academic performance and leadership. We find that Poet vs. Quant aptitudes and interests uniquely predict respective performance. GMAT-Quantitative predicted performance in “quant” classes, and GMAT-Verbal predicted performance in “poet” classes. Interestingly, GMAT-Verbal also predicted performance in “quant” courses, likely because even numeric judgments need to be conveyed verbally and socially in the classroom. GMAT-Verbal and GMAT-Writing also predict leadership performance, although in different respects that speak to prior debates in management education research about their relevance.

Third, our work supports the usefulness of the GMAT-Writing in predicting meaningful outcomes. While some prior work questions the predictive usefulness of GMAT-W (Sireci and Talento-Miller, 2006), more recent studies have found that it does predict leadership performance (Kass et al., 2012). We provide supporting evidence for this in Study 2 where we found a positive relationship between GMAT-Writing and objective leadership positions attained. This supports an argument that GMAT-Writing scores deserve more weight in the admissions process. However, peer-perceived leadership was not predicted by GMAT-Writing but instead by GMAT-Verbal. The inconsistency among these findings and those of Kass et al. highlights the need for further empirical research with a broader set of subjective and objective leadership indicators.

A fourth contribution is the development and validation of additional social measures of MBA performance (club leadership and classmate appraisals). While prior work typically operationalizes MBA success through grades, our Study 2 highlights that additional real world social outcome variables can and should be measured. These measures deserve increased attention due to their importance when selecting for leadership roles.

### 5.2 The evolving art of assessing interests

A fifth contribution is strong support for the relevance of student interests as predictors of MBA outcomes, highlighting the importance of this often-neglected individual difference construct. Interests were predictive over and above aptitudes in Study 2. Specifically, quantitative general interests also predicted performance in quantitative courses, although the relationships were less clear with respect to interests in particular industry domains. We did not find that verbal interests predicted performance in social courses. Both quantitative and social interests predicted objective leadership performance—the former negatively, and the latter positively—although the pattern for social interests varied depending on how interests were measured.

Our measure of students’ general interests was, overall, more predictive than our measure of their post-MBA career interests. The general interests measure, CareerLeader, consists of self-ratings on dozens of distinct activities, and descends from a long tradition of vocational interest inventories in industrial-organizational psychology (Campbell and Borgen, 1999; Campbell et al., 1974; Strong, 1943). We suspect that this survey-based measure accurately captures internal preferences while the admissions application measure of industry goals might reflect additional factors like social or financial pressures.

The student self-interest assessments in our study were collected in the context of career counseling, not admissions, so self-presentation influences were low. However, such measures may be subject to impression management and self-report biases if known to be used in admissions decisions. Emerging trends in assessment, such as the reliance on digital traces, (e.g., Kosinski et al., 2015; Kulkarni et al., 2018) may provide alternate operationalization of interests that are not subject to self-report concerns.

### 5.3 Admissions office folklore

A sixth major contribution of this paper is that it tested a commonly held belief among some admissions officers that varsity sports experience and past military service are relevant to future MBA and leadership success. We did not find any evidence that such experience predicts either academic or leadership success within the MBA program. This raises the question of what underlies the admissions officers’ beliefs and whether some other measure detectable at admissions time is coincident with such experiences.

### 5.4 Limitations

Our data were sourced from an elite business school in the northeastern United States. Although we are confident—given common applicant pools and employers—that the results would generalize to students at peer institutions, we do not know if the results would generalize across the full spectrum of business schools serving different student populations. We note shortcomings of ethnic and gender representation in our student population, representative of top US MBA programs at the time. Related research on this population has found effects on academic and leadership performance of gender (Wallen et al., 2017) and cultural backgrounds (Lu et al., 2020; Lu et al., 2022).

In addition to social intelligence, research on internationally diverse settings increasingly also measures cultural intelligence (Erez et al., 2013). These scales capture individual strengths, such as cultural metacognition, that predict intercultural trust and collaboration (Chua et al., 2012). For international students at US business schools, part of the challenge in academic and leadership success is learning the implicit cultural norms. Cultural metacognition predicts this learning curve (Morris et al., 2019) and standard measures of cognitive intelligence or IQ do not (Savani et al., 2022). Future research can investigate whether cultural intelligence measures add predictive validity beyond social intelligence measures in predicting MBA success and whether this is especially so for international students.

Likewise, it is unclear if these results would generalize to schools in other countries, where cultural norms may suggest other measures of leadership performance, for example. It is also unclear if GMATs would predict validly across cultures as well. A case study from a university located in the Middle East suggests that GMATs may not be viewed favorable in at least one non-U.S. cultural context (Al-Badi et al., 2008). In addition, other research suggests that GMAT scores are influenced by national culture, and that they may negatively predict measures of ethnical qualities (Aggarwal et al., 2013). However, the population we studied has large international representation and we control for US citizenship, reducing concerns that the specific location of the school impacts generalizability.

### 5.5 Future directions

We have provided evidence that MBA success can be categorized into distinct poet and quant domains. These findings prompt many further lines of inquiry relevant to various stakeholders, including admissions officers, curriculum designers, students, and even hiring managers. One future research stream might focus on whether admissions officers should look for candidates who are competent in both domains or those that simply meet some threshold criteria in one domain but are truly extreme in the other. Imagine an admissions officer is presented with two candidates with the same average poet and quant scores – yet one is average on both and the second is mediocre on one yet exceptional on the other. Which of these two profiles would lead to greater success in school and in the workplace? Best practices in this regard have implications for the resulting makeup of the MBA cohort and the potential for achieving a more diverse and well-rounded group of students. A recent finding suggests that emotional intelligence scores can interact with cognitive scores in predicting MBA student performance (Truninger et al., 2018).

A second line of inquiry might focus on the generalization or specialization of MBAs in either the poet or quant domains. Are students better off being a “renaissance person” or “jack of all trades” through building up competencies in their less preferred domain? Or are they better off forgetting about their weaknesses and building on their strengths? These questions have implications for individual MBA students as well as for programs administrators and curriculum designers. Given the nature of teams and teamwork in the workplace, students now often have the option to benefit from complementing their strengths with those of others, and thus may not need to have certain capabilities themselves. This might argue for fewer required classes and more flexibility in pursuing individual interests. This is also relevant for schools focusing on more quantitative disciplines such as accounting or more social disciplines such as marketing.

Although we have focused on MBA student success and the predictors used in the admissions process, there are also relevant parallels to employee selection. Future work can focus on how hiring and selection may be optimized or tailored for different manifestations of professional success. After all, any well-designed talent acquisition process requires a well-defined success criterion. Our findings make a case for identifying distinct success criteria beyond those that are merely technical. For example, competencies in working with people become increasingly important as employees ascend into managerial and leadership roles. Hiring procedures that incorporate predictors of ‘poet’ skillsets and interests may filter more promotable employees than procedures that screen on unidimensional quantitative abilities (Smith et al., 2020; Liu and Boyatzis, 2021). Identification of relevant selection-time predictors could lead to more efficient and effective hiring and search processes. In addition to selection researchers, our findings are a call to action for hiring managers and human resources executives, who might be well-served in adopting a more expansive list of selection procedures, with an eye toward identifying success in its multiple forms.

## Data Availability

The data analyzed in this study is subject to the following licenses/restrictions: underlying data for both studies are not publicly available due to its confidential nature, the U.S. ‘FERPA’ law against divulging student grades, lack of IRB approval for public disclosure, and explicit assurances to multiple university offices involved that their proprietary data would not be publicly released as a condition of data sharing. We are, of course, happy to perform any additional analysis requested. Requests to access these datasets should be directed to aw2328@columbia.edu.

## Footnotes

1 The current paper does not intend to purposefully claim or validate elite status for particular institutions and rather takes as given the current status hierarchy as reflected by commonly publicized ranking systems.

2 Integrated reasoning has since been added as a fourth section.

3
http://www.careerleader.com
